# Comparison of facilities with and without additional medical fees for nutrition support team activity during the COVID-19 pandemic

**DOI:** 10.1186/s40780-024-00389-z

**Published:** 2024-10-31

**Authors:** Akihiko Futamura, Takenao Koseki, Junichi Iida, Akito Suzuki, Nobuyuki Muroi, Michiaki Myotoku, Hiroki Maki, Kazuhisa Mizutani, Hikaru Ogino, Yasuki Taniguchi, Keiichiro Higashi, Masanobu Usui

**Affiliations:** 1 Department of Pharmacy, Fujita Health University Nanakuri Memorial Hospital, 424-1, Oodori, Tsu, Mie 514-1295 Japan; 2 Department of Pharmacotherapeutics and Informatics, Fujita Health University School of Medicine, 1-98, Dengakugakubo, Kutsukake-Cho, Toyoake, Aichi 470-1192 Japan; 3 Hospitalization Support Center, Saiseikai Yokohama-Shi Nanbu Hospital, 3-2-10, Konandai Konan-Ku, Yokohama, Kanagawa 234-0054 Japan; 4 Department of Pharmaceutical Sciences, School of Pharmaceutical Sciences, Kyushu University of Medical Science, 1714-1 Yoshinomachi, Nobeoka, Miyazaki 882-8508 Japan; 5 Department of Pharmacy, Kobe City Medical Center General Hospital, 2-1-1, Minatojima Minamimachi, Chuo-Ku, Kobe 650-0047 Japan; 6 Faculty of Pharmacy, Osaka Ohtani University, 3-11-1, Nishikioritaki, Tondabayashi, Osaka 584-8540 Japan; 7 Department of Pharmacy, Kofu City Regional Medical Center, 1-18-1 Marunouchi, Kofu, Yamanashi 400-8585 Japan; 8 Department of Pharmacy, Toya Onsen Hospital, 54-41 Abutagun Toyakocho, Hokkaido, 049-5892 Japan; 9 Tokai Pharmacy, Tokai Pharmacy in Front of Nakatsugawa Municipal Hospital, 1666-1152 Komanbacho, Nakatsugawa, Gifu 508-0011 Japan; 10 Department of Pharmacy, Inabe General Hospital, 771 Hokuseichouageki, Inabe, 511-0428 Japan; 11 Department of Pharmacy, Asanogawa General Hospital, 83 Kosakamachinaka, Kanazawa, Ishikawa 920-8621 Japan; 12 Department of Surgery & Palliative Medicine, Fujita Health University School of Medicine, Toyoake, 470-1192, Japan

**Keywords:** Central line-associated bloodstream infection, Nutrition support team, COVID-19 pandemic, Additional medical fee for the nutrition support team activity, Pharmacy

## Abstract

**Background:**

This study aimed to clarify the effectiveness of nutrition support team (NST) facilities for preventing central line-associated bloodstream infection (CLABSI).

**Methods:**

We retrospectively analyzed the incidence of CLABSI as well as the presence or absence of additional medical fees for NST activity between 2019 and 2021, including the period before and after the COVID-19 pandemic. Subsequently, we performed between-group comparisons of the CLABSI incidence. CLABSI rates were compared based on cumulative per 1000 catheter uses during the relevant period.

**Results:**

Among 47 facilities that were registered for participation, there were 34 and 13 facilities with and without additional medical fees for NST activity (NST and non-NST groups, respectively). The CLABSI incidence rate was significantly lower in the NST group 0.96 [0.28–1.73] than in the non-NST group 1.25 [075–6.10] (*p* < 0.05). Before the pandemic, the NST group had a lower CLABSI rate per 1000 catheter uses than the non-NST group 2019: 0.70 [0.12–1.26] vs 2.10 [0.62–5.97]. During the pandemic, the CLABSI incidence showed no significant between-group difference 2020: 0.99 [0.51–1.61] vs 1.01 [0.80–4.16]; 2021: 1.24 [0.44–2.35] vs 1.96 [1.23–5.31]; however, the CLABSI rates in the NST group remained low.

**Conclusion:**

During the COVID-19 pandemic, the incidence of CLABSI was lower in the NST group than in the non-NST group, indicating the effectiveness of NST in preventing the occurrence of CLABSI.

## Background

Catheter-related bloodstream infections (CRBSIs) are the most common healthcare-associated infections. Worldwide, there has been extensive research on central line-associated bloodstream infections (CLABSIs) specifically, as they have been associated with extended duration of hospital stay, high mortality rates, and increased medical costs [1–5]. In the United States, with the development and adaption of central parenteral nutrition, there has been a concomitant need to suppress related complications such as CLABSI; accordingly, the nutrition support team (NST) was established [6, 7]. In Japan, the NST has been popularized through the initiative of the Japan Society for Nutritional Therapy. Further, in the 2010 medical fee revision, NST facilities were defined as those with an additional medical fee for NST activity. The NST comprises specialized doctors, nurses, pharmacists, and registered dietitians who attend to patients with malnutrition, blood albumin levels ≤ 3.0 g/dL, and a need for enteral or parenteral nutrition. The NST holds meetings and conferences to promote recovery and prevent complications such as infectious diseases. In addition, it holds joint conferences and collaborates with infection control teams. However, within the > 10-year period following the establishment of the NST, there have been very few nationwide studies on the effects of the NST on the incidence of CLABSI and parenteral nutrition management. Moreover, although the COVID-19 pandemic has affected various functions within medical institutions [8–10], the effect of the NST on the incidence of CLABSI during the COVID-19 pandemic remains unclear. Accordingly, this study aimed to investigate the effectiveness of NST in preventing the occurrence of CLABSI before and during the COVID-19 pandemic.

## Materials and methods

### Study design and population

This retrospective observational study included hospitalized patients diagnosed with CLABSI between January 2019 and December 2021. The primary endpoint was the incidence of CLABSI per 1000 catheters used; additionally, we compared the incidence of CLABSI between the NST and non-NST groups. CLABSI rates were compared based on cumulative per 1000 catheter uses during the relevant period.

The facility standard for infection control prevention additional medical fee 1 (ICP-1) is to have a full-time in-hospital infection control staff and an infection prevention department. An infection prevention team consisting of full-time doctors, nurses, pharmacists, and laboratory technicians is organized to carry out daily infection prevention work. The facility standard for infection control prevention additional medical fee 2 (ICP-2) is a facility with fewer than 300 general hospital beds. An infection prevention team consisting of full-time doctors or nurses is organized.

### Data collection

We invited pharmacists who were members of the Japanese Society for Parenteral and Enteral Nutrition Therapy to participate in the study through the society's official website and direct mail. Data were collected using an electronic data collection system. The survey items included the type of medical facility, number of beds, number of patients with CLABSI, duration (days) of central venous catheter placement, ICP-1 and 2, CRBSI prevention manual and its documentation, sterile preparation processing fee, sterile preparation practices for total and peripheral parenteral nutrition, use of closed-system infusion lines and in-line filters, selection of disinfectants for central venous catheter insertion sites, and saline flush volume following fat emulsion administration.

The CRBSI prevention manual includes hand hygiene, indications and selection of catheter use, appropriate insertion method, in-line filters, infusion management method, CRBSI risk, and education for staff [11]. Among these, the principles for high-calorie infusion include choosing enteral nutrition whenever possible and mixing drugs into high-calorie infusion preparations in a sterile environment in the pharmacy whenever possible. Catheters are classified into short-term placement, long-term placement (Broviac catheter, Hickman catheter), completely subcutaneously implanted, and PICC (Peripherally inserted central venous catheter), and are selected according to the purpose of use. When inserting a central venous catheter, it is recommended that the insertion site be disinfected with chlorhexidine containing alcohol at a concentration of more than 0.5% and that maximal barrier precautions be used. In addition, since lipid emulsions and blood products have been identified as independent risk factors for CRBSI, the manual includes regular replacement of infusion sets and flushing methods for infusion lines.

### Statistical analysis

Statistical analyses were performed using EZR (Saitama Medical Center, Jichi Medical University, Saitama, Japan) as a graphical user interface for R version 4.2.2 (The R Foundation for Statistical Computing, Vienna, Austria). EZR, a modified version of R Commander (version 1.61), is designed to incorporate commonly used statistical functions in biostatistics [12]. Continuous variables were expressed as medians and ranges, and categorical variables were summarized as frequencies and proportions. Between-group comparisons of quantitative and qualitative data were performed using the Mann–Whitney test, respectively. Statistical significance was set at *p* < 0.05.

## Results

### Facility information

There were 47 registered facilities (33 general hospitals, 10 mixed healthcare facilities, 3 nursing facilities, and 1 psychiatric hospital); among them, 34 and 13 facilities were in the NST and non-NST groups, respectively. Additionally, 85.3% and 53.8% of facilities in the NST and non-NST groups were general hospitals and mixed healthcare facilities, respectively. Regarding the number of beds, the NST group included large hospitals with > 600 beds while 30.8% of facilities in the non-NST group had < 200 beds. Further, 79.4% and 46.2% of facilities in the NST and non-NST groups, respectively, met the facility standards for ICP-1. Table 1 shows other facility information related to CLABSI.

**Table 1:** Facility information

	Total *n* = 47	NST group *n* = 34	Non-NST group *n* = 13
National hospital	2 (4.3)	1 (3.0)	1 (7.7)
Public hospital	18 (38.3)	15 (44.1)	3 (23.1)
Private medical institutions	27 (57.4)	18 (52.9)	9 (69.2)
General hospitals	33 (70.2)	29 (85.3)^*^	4 (30.8)
Mixed healthcare facilities	10 (21.3)	3 (8.8)	7 (53.8)
Nursing facilities	3 (6.4)	2 (5.9)	1 (7.7)
Psychiatric hospital	1 (2.1)	0 (0.0)	1 (7.7)
< 200 beds	8 (17.0)	4 (11.8)	4 (30.8)
200–599 beds	30 (63.8)	21 (64.7)	9 (69.2)
> 600 beds	9 (19.2)	9 (26.5)	0 (0.0)
Facilities for infection control prevention additional medical fee 1	33 (70.2)	27 (79.4)^**^	6 (46.2)
Facilities for infection control prevention additional medical fee 2	14 (29.8)	7 (20.6)	7 (53.8)
Presence of standards such as CRBSI prevention manuals			
Standards ( +) / Documentation ( +)	41 (87.2)	31 (91.1)	10 (76.9)
Standards ( +) / Documentation (-)	5 (10.6)	2 (5.8)	3 (23.1)
Standards (-) / Documentation (-)	1 (2.1)	1 (2.9)	0 (0.0)
Number of facilities with sterile preparation processing fee/non-fee (%)	35/12 (74.5/25.5)	27/7 (79.4/20.6)	8/5 (61.5/38.5)
TPN sterile preparation rate	80 [57–95]	80 [70–90]	83 [31–98]
PPN sterile preparation rate	0 [0–5]	0 [0–5]	0 [0–38.7]
Number of closed-infusion systems used	44 (93.6)	31 (91.1)	13 (100.0)
Number of inline filters used	28 (59.5)	18 (52.9)	10 (76.9)
Disinfectant for CVC insertion site			
Povidone–iodine	33 (70.2)	25 (73.5)	8 (61.5)
Chlorhexidine–alcohol	21 (44.6)	17 (50.0)	4 (30.7)
Ethanol	8 (17.0)	7 (20.5)	1 (7.6)
Chlorhexidine	6 (12.7)	4 (11.7)	2 (15.3)
Flush volume following fat emulsion administration			
5 mL saline	5 (10.6)	4 (11.7)	1 (7.6)
10 mL saline	21 (44.6)	14 (41.1)	7 (53.8)
20 mL saline	15 (31.9)	10 (29.4)	5 (38.4)
50 mL saline	2 (4.2)	2 (5.8)	0 (0.0)

% (number), Median [interquartile range]

^*^p < 0.01

^**^p < 0.05

### Incidence of CLABSI

**Fig. 1:**
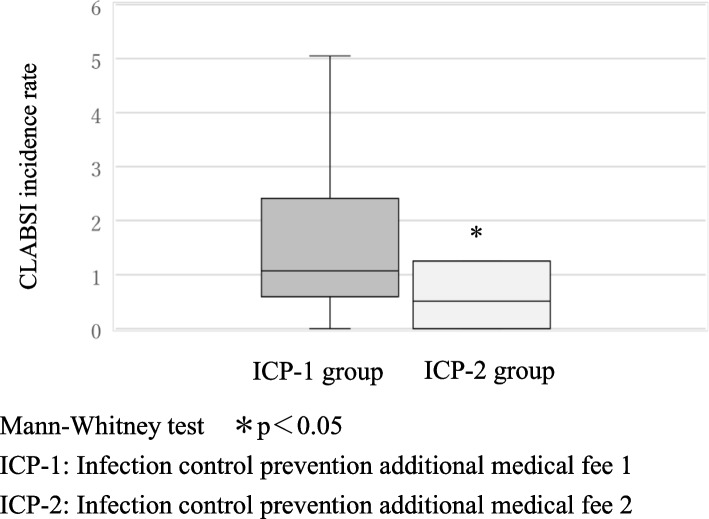
CLABSI incidence rate per 1000 catheter uses in ICP-1 and ICP-2 groups (Over a 3-year period from 2019 to 2021)

**Fig. 2:**
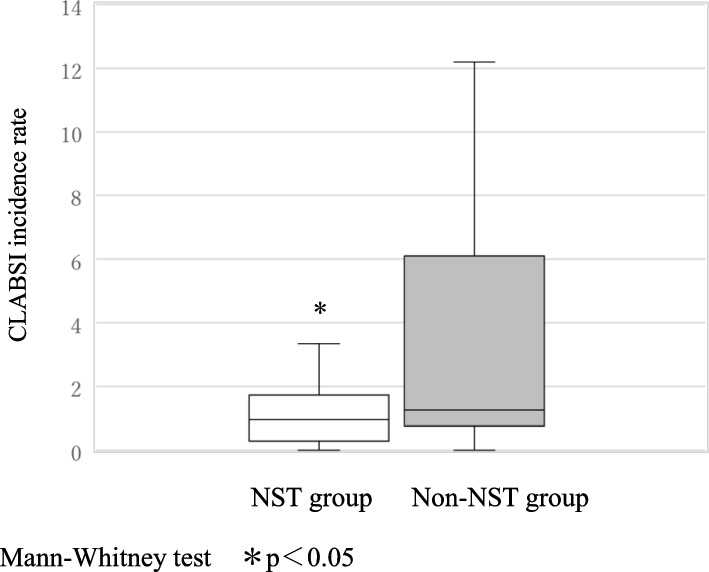
CLABSI incidence rate per 1000 catheter uses in NST and non-NST groups (Over a 3-year period from 2019 to 2021)

**Fig. 3:**
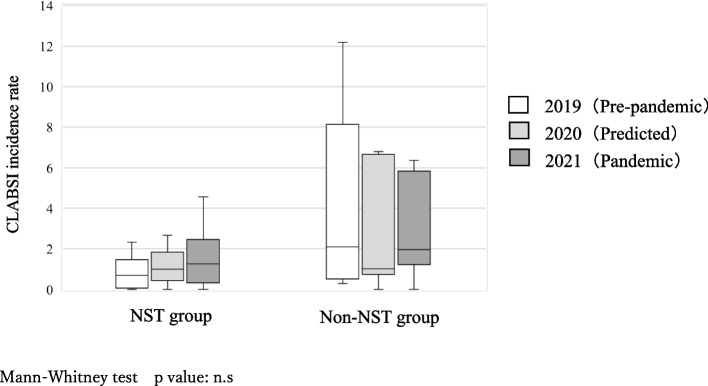
CLABSI incidence rate per 1000 catheter uses in NST and non-NST groups (before and during the pandemic)

Over a 3-year period between 2019 and 2021, the CLABSI incidence rate per 1000 catheter uses was significantly lower in the ICP-2 group 0.50 [0.00—1.24] than in the ICP-1 group 1.07 [0.60—2.31], *p* < 0.05, (Fig. 1). Over a 3-year period between 2019 and 2021, the CLABSI incidence rate per 1000 catheter uses was significantly lower in the NST group 0.96 [0.28—1.73] than in the non-NST group 1.25 [0.75—6.10], *p* < 0.05, (Fig. 2). The overall CLABSI incidence per 1000 catheter uses was 0.70 [0.29—1.82] in 2019, 1.00 [0.52—1.73] in 2020, and 1.32 [0.55—2.59] in 2021. Before the pandemic, the NST group had a lower CLABSI rate per 1000 catheter uses than the non-NST group 2019: 0.70 [0.12—1.26] vs 2.10 [0.62—5.97]. During the pandemic, the CLABSI incidence showed no significant between-group difference 2020: 0.99 [0.51—1.61] vs 1.01 [0.80—4.16]; 2021: 1.24 [0.44—2.35] vs 1.96 [1.23—5.31]; however, the CLABSI rates in the NST group remained low (Fig. 3).

## Discussion

This study investigated the effectiveness of NST in preventing the occurrence of CLABSI before and during the COVID-19 pandemic.

Implementation of the NST has been associated with improved survival outcomes in critically ill patients with COVID-19 [13]. However, there have been no reports focusing on CLABSI and no studies on the NST in Japan. In Japan, the NST provides multi-disciplinary nutritional management to patients with malnutrition or those at risk of malnutrition to promote the healing of underlying diseases and to prevent complications such as infections. The training of healthcare professionals on infection prevention manuals for central venous catheter management is effective in reducing device-standardized infection rates and preventing CLABSI [14]. In the NST group, the documentation of the standards of the CLABSI prevention manual and the establishment of a system for implementing aseptic preparation could have contributed to the suppression of CLABSI occurrence. In our study, 93.6% and 59.5% of facilities used closed infusion systems and in-line filters, respectively, with no significant between-group difference. The use of antiseptics on central venous catheter insertion sites has demonstrated clinical preventive effects; among them, chlorhexidine–alcohol significantly reduces the incidence of CLABSI compared with povidone–iodine [15]. In addition, current guidelines recommend alcohol with chlorhexidine added at a concentration of > 0.5% [16]. Among the target facilities, povidone–iodine was the most widely used antiseptic. Chlorhexidine-induced anaphylaxis has been reported to be more common in the Japanese population than in the Caucasian population [17]. Accordingly, 10% povidone–iodine should be considered an option for skin disinfection; however, education on its usage is important. Fat emulsion administration is a risk factor for CLABSI [18]. Specifically, following fat emulsion administration, microorganisms that have entered the intravenous infusion line may proliferate over time. Therefore, it is important to flush the line with a sufficient saline volume to prevent CLABSI development. In both groups, the saline flush volume after fat emulsion administration was ≤ 10 mL in more than half of the patients. The required saline flush volume may be approximately twice the capacities of the indwelling needle, catheter, and connected device. Taken together, the administration of fat emulsions that promote microbial growth is an independent risk factor for CRBSI; therefore, it should be followed by an appropriate saline flush volume and frequent infusion set changes [19, 20].

During the COVID-19 pandemic, there was postponement or cancellation of elective surgeries as well as a need for an increased number of intensive care unit beds. Additionally, patients with COVID-19 have shown an increased incidence of various healthcare-associated infections, comorbidities, and long-term hospitalization [21, 22]. In our study, the incidence of CLABSI after the pandemic was non-significantly higher than that before the pandemic, which is consistent with previous findings [21, 22]. In 2020, the CLABSI incidence rate increased and decreased in the NST and non-NST groups, respectively. Before the pandemic, teamwork contributed to reducing the infection rate. During the pandemic, many facilities in the NST group were classified as Infection Control and Prevention Level 1; additionally, they received an increased number of patients since they accepted critically ill patients, which required targeted measures. Accordingly, during the pandemic, team activities were impeded and became stagnant. According to the Ministry of Health, Labour and Welfare's report on "Number of medical institutions accepting COVID-19 patients by national, public, and private institutions and the percentage of acceptances" as of November 2020, the acceptance rates of COVID-19 patients at national and public medical institutions are high at 58% and 75%, respectively, while the rate at private medical institutions is low at 17% [23]. This study was not able to collect data on the acceptance of COVID-19 patients at participating facilities. However, as shown in Table 1, we speculated that the restriction on the admission of COVID-19 patients may have been one of the factors that reduced the incidence of CLABSI since the non-NST group had a higher proportion of private medical institutions than the NST group.

This study has several limitations. First, it was a retrospective observational study, and we could not adjust for all confounding factors affecting the incidence of CLABSI.

Second, although there are numerous NST facilities operating nationwide, we included a small number of facilities which limits generalizability. Third, the incidence of CLABSI may be affected by various factors, including the infection control system, and further study is needed to prove the effect is limited to NST. Multi-center studies accounting for these limitations are warranted to focus on the coordination system of medical teams such as the NST and infection control teams. Elucidating the effectiveness of the NST in preventing the occurrence of CLABSI could inform the improvement of the NST activity with respect to the fight against emerging and unknown infections.

In our study, the incidence of CLABSI during the COVID-19 pandemic was lower in the NST group than in the non-NST group, which indicates the effectiveness of the NST in preventing the occurrence of CLABSI.

## Data Availability

Data and materials are available through Fujita Health University Research Electronic Data Capture.

## Abbreviations

NST: Nutrition support team
CLABSI: Central line-associated bloodstream infection
COVID-19: Coronavirus disease
ICP: Infection control prevention additional medical fee
