# Correction: Comparison of facilities with and without additional medical fees for nutrition support team activity during the COVID-19 pandemic

**DOI:** 10.1186/s40780-024-00407-0

**Published:** 2025-01-08

**Authors:** Akihiko Futamura, Takenao Koseki, Junichi Iida, Akito Suzuki, Nobuyuki Muroi, Michiaki Myotoku, Hiroki Maki, Kazuhisa Mizutani, Hikaru Ogino, Yasuki Taniguchi, Keiichiro Higashi, Masanobu Usui

**Affiliations:** 1 Department of Pharmacy, Fujita Health University Nanakuri Memorial Hospital, 424-1, Oodori, Tsu, Mie, 514-1295 Japan; 2 Department of Pharmacotherapeutics and Informatics, Fujita Health University School of Medicine, 1- 98, Dengakugakubo, Kutsukake-Cho, Toyoake, Aichi 470-1192 Japan; 3 Hospitalization Support Center, Saiseikai Yokohama-Shi Nanbu Hospital, 3-2-10, Konandai Konan-Ku, Yokohama, Kanagawa 234-0054 Japan; 4 Department of Pharmaceutical Sciences, School of Pharmaceutical Sciences, Kyushu University of Medical Science, 1714-1 Yoshinomachi, Nobeoka, Miyazaki 882-8508 Japan; 5 Department of Pharmacy, Kobe City Medical Center General Hospital, 2-1-1, Minatojima Minamimachi, Chuo-Ku, Kobe, 650-0047 Japan; 6 Faculty of Pharmacy, Osaka Ohtani University, 3-11-1, Nishikioritaki, Tondabayashi, Osaka 584-8540 Japan; 7 Department of Pharmacy, Kofu City Regional Medical Center, 1-18-1 Marunouchi, Kofu, Yamanashi, 400- 8585 Japan; 8 Department of Pharmacy, Toya Onsen Hospital, 54-41 Abutagun Toyakocho, Hokkaido, 049-5892 Japan; 9 Tokai Pharmacy, Tokai Pharmacy in Front of Nakatsugawa Municipal Hospital, 1666-1152 Komanbacho, Nakatsugawa, Gifu, 508-0011 Japan; 10 Department of Pharmacy, Inabe General Hospital, 771 Hokuseichouageki, Inabe, 511-0428 Japan; 11 Department of Pharmacy, Asanogawa General Hospital, 83 Kosakamachinaka, Kanazawa, Ishikawa 920- 8621 Japan; 12 Department of Surgery & Palliative Medicine, Fujita Health University School of Medicine, Toyoake, 470- 1192 Japan

**Journal of Pharmaceutical Health Care and Sciences (2024) 10:67**

10.1186/s40780-024-00389-z

The original publication of this article contained an incorrect acknowledgement section. The incorrect and correct information is shown in this correction article. The original article has been updated.

**Incorrect**

We thank Tomomi Jinnouchi in Makino Rehabilitation Hospital; Naohito Nakamura in Tosei General Hospital; Mizuho Kabeya in Fuefuki Central Hospital; Takaki Kanie in the Department of Pharmacotherapeutics and Informatics, Fujita Health University hospital; Sayaka Taji in Minoh City Hospital; Fumie Yamada in Tsukuba Medical Center Hospital; Takeshi Yoshimi in Japanese Red Cross Medical Center; Toshihiro Nakanishi in Clinical Safety Management Group, Toyota Memorial Hospital, Toyota Motor Corporation; Masaya Ito in Hashima City Hospital; Mayu Shibata in Hamamatsu Red Cross Hospital; Hiroshi Ishihara in the Department of Pharmacy, Japanese Red Cross Gifu Hospital; Hiroko Kimbara in Public Central Hospital of Matto Ishikawa; Shin Inoue in Oita Oka Hospital; Keisuke Suzuki in Taito Hospital; Masashi Fujita in Komono Kosei Hospital; Namiki Makiko in Funabashi Municipal Medical Center; Yukari Takeno in Oota Hospital; Yoshihiro Uekuzu in the Department of Pharmacy, Fujita Health University Okazaki Medical Center; Yuka Aimono in Hitachi General Hospital; Maho Tatsumi in Kakogawa Central City Hospital; Takashi Matsuzaki in the Department of Pharmacy, St. Marianna University, Yokohama Seibu Hospital; Hiroshi Shinonaga in the Department of Pharmaceutical Services, Mitoyo General Hospital; Taku Ito in the Department of Pharmacy, Tenshi Hospital; Miki Kawasaki in Neuromuscular Center Yoshimizu Hospital; Yoichi Shibata in National Hospital Organization Yamagata Hospital; Hasegawa Mio in Fujiikai Kashibaseiki Hospital; Akiko Sasaki in Hibino Hospital; and Kyouko Mori in Fureai-Kamakura Hospital for providing data regarding patients with CRBSI. The authors thank Editage (https://www.editage.com/) for editing and reviewing the manuscript for the English language. This work was supported by the Japanese Society for Clinical Nutrition and Metabolism.

**Correct**

We thank Tomomi Jinnouchi in Makino Rehabilitation Hospital; Naohito Nakamura in Tosei General Hospital; Mizuho Kabeya in Fuefuki Central Hospital; Rie Nishida and Takaki Kanie in, Fujita Health University hospital; Sayaka Taji in Minoh City Hospital; Fumie Yamada in Tsukuba Medical Center Hospital; Takeshi Yoshimi in Japanese Red Cross Medical Center; Toshihiro Nakanishi in Clinical Safety Management Group, Toyota Memorial Hospital, Toyota Motor Corporation; Masaya Ito in Hashima City Hospital; Mayu Shibata in Hamamatsu Red Cross Hospital; Hiroshi Ishihara in the Department of Pharmacy, Japanese Red Cross Gifu Hospital; Hiroko Kimbara in Public Central Hospital of Matto Ishikawa; Shin Inoue in Oita Oka Hospital; Keisuke Suzuki in Taito Hospital; Masashi Fujita in Komono Kosei Hospital; Namiki Makiko in Funabashi Municipal Medical Center; Yukari Takeno in Oota Hospital; Yoshihiro Uekuzu in the Department of Pharmacy, Fujita Health University Okazaki Medical Center; Yuka Aimono in Hitachi General Hospital; Maho Tatsumi in Kakogawa Central City Hospital; Takashi Matsuzaki in the Department of Pharmacy, St. Marianna University, Yokohama Seibu Hospital; Hiroshi Shinonaga in the Department of Pharmaceutical Services, Mitoyo General Hospital; Taku Ito in the Department of Pharmacy, Tenshi Hospital; Miki Kawasaki in Neuromuscular Center Yoshimizu Hospital; Yoichi Shibata in National Hospital Organization Yamagata Hospital; Hasegawa Mio in Fujiikai Kashibaseiki Hospital; Akiko Sasaki in Hibino Hospital; Hideki Yamaguchi in U Yakkyoku; Kyoko Saito in Kakio Memorial Hospital; Shigeki Ikushima in Nara Prefecture General Medical Center; Hitoshi Yagi in Kanto Rosai Hospital; Eiji Hotta in Fukui-ken Saiseikai Hospital; Yusuke Takata in Ehime University Hospital; Hiroki Asakawa in Hokuto Municipal Koyo Hospital; Yoji Ito in Saiki Central Hospital; Mariko Nakamichi in Haradoi Hospital; Takuya Sugahara in Yamagata City Hospital Saiseikan; Daisuke Maekawa in Ikoma City Hospital; Yoshikazu Watanabe in Saiseikai Utsunomiya Hospital; Tsukasa Sekimoto in Hasegawa Hospital; and Kyouko Mori in Fureai-Kamakura Hospital for providing data regarding patients with CRBSI. The authors thank Editage (https://www.editage.com/) for editing and reviewing the manuscript for the English language. This work was supported by the Japanese Society for Clinical Nutrition and Metabolism.

